# A new understanding of health related empowerment in the context of an active and healthy ageing

**DOI:** 10.1186/s12913-019-4082-5

**Published:** 2019-04-24

**Authors:** Lars Kayser, Astrid Karnoe, Emily Duminski, David Somekh, Cecilia Vera-Muñoz

**Affiliations:** 1 Department of Public Health, Faculty of Health and Medical Sciences, University of Copenhagen, Copenhagen, Denmark; 2 European Health Futures Forum, Dromahair, Ireland; 3 Life Supporting Technologies, ETSI Telecomunicación, Universidad Politécnica de Madrid, Madrid, Spain

**Keywords:** Health-related empowerment, delphi, active ageing, chronic conditions

## Abstract

**Background:**

Recently, several initiatives have focused on how to create true person-centred health services. This calls for a new understanding of health-related empowerment in relation to people living with one or more chronic conditions.

We report on a Delphi investigation among participants in the European Innovation Partnership on Active and Healthy Ageing that has led to a new understanding of health-related empowerment.

**Methods:**

The Delphi process was conducted in three sequential rounds. In the first round, we presented a suggested first version for a definition of “health-related empowerment” divided into nine statements. One hundred and twenty-two experts were then asked if they agreed or not with each individual statement, and in the case they disagreed, to state the reasons for their disagreement.

After revisions, the experts who had replied to the first version were asked again, if they agreed or not with each individual statement of the second version and to elaborate on disagreements.

Finally, in the third round the experts were asked to provide comments to the final proposed definition in general and not by each statement.

**Results:**

A total of 33 experts responded to the first version. The following revision included a merging of two statements, and the addition of health literacy as part of the understanding. The second version was sent out to the 33 experts and a total of 19 experts commented with moderate consensus. Changes included removal of “self-esteem” and change of “self-confidence” to confidence.

Third version was sent out to all 122 experts with 16 respondents. Strong consensus was obtained for this third version, and is with one minor change presented as the final version.

**Conclusion:**

We propose a new understanding of the concept health-related empowerment, by focusing on the individual as a co-manager with freedom to choose and focus on their own well-being.

## Background

Since the publication of the Chronic Illness Model by Ed Wagner in 1996 [1] there has been an increased interest in how people living with one or more chronic conditions can be supported in and enabled to manage their health condition, also in collaboration with formal and informal caregivers. Wagner was inspired by the abundant literature from the second half of the twentieth century pointing to the importance of active involvement of individuals in their condition. In the second half of the twentieth century, the concept of empowerment developed and influenced new ways of thinking also in the context of health.

The 1960’s “power to” movement had influenced health professionals’ practice. As a reaction to the prevailing paternalism in health care, there were as early as the beginning of the 1960s academics that challenged the prevailing social order in alignment with the general tendencies in society [2]. The key elements here were the principles of choice over issues that affect one’s life, autonomy and pro-active involvement.

In the wider social sphere the “power to the people” movements materialised in the pursuit of social justice and civil rights. Movements included the Black Power Movement and women’s liberation [3]. These movements led to an increasing awareness of the importance of a shift towards a bottom-up process giving voice to the people. This development in society is reflected in Arnstein’s introduction of The Ladder of Citizen Participation in 1969 [4]. The ladder illustrates different steps or levels of citizen involvement and acts as a guide to understand the power balance in each of these steps. In the 1970’s, healthcare started moving from a disease-centred model towards a more person-centred model and this challenged the concept of the passive patient [5].

Consequently, in 1986 the Ottawa Charter introduced the concept of empowerment in relation to health promotion [6]. The Ottawa Charter highlighted the important notion that “empowerment” is both about the individual but also refers to the organisational level. The process towards empowerment begins with participation of the individual, which leads to ownership with the ultimate goal of giving the individual control over own condition [6].

Since the Ottawa Charter, the concept of patient empowerment has developed in many directions and several stakeholders have approached the concept differently [3, 7–10]. This may be a result of the various stakeholders’ position in the health care system. At the micro level stakeholders focus on the individual’s needs, preferences and competence which may be influenced by socio-demographic factors, the cultural context, and past experiences. At the meso level the concept of patient empowerment is influenced by organisational norms and values. At the macro level the political culture, meaning the power relations between the individual and the state, influences the understanding of empowerment.

In 1995 Zimmermann proposed a measureable concept of psychological empowerment [11]. This construct may have a broader application than the area of health, but is relevant to the description of health-related empowerment. According to Zimmermann, the construct of psychological empowerment is an integration of perceptions of personal control, a proactive approach to life, and a critical understanding of the socio-political environment. Psychological empowerment should be understood as a dynamic process that differs across time, context, and between individuals [11]. To be psychologically empowered an individual should have the capability to influence a given context (intrapersonal component), be able to understand how the system works in a certain context (interactional component), and engage in behaviours to exert control in the context (behavioural component) [11].

In 2013, Schulz and Nakamoto identified four elements to be addressed to empower patients based on previous literature [12]. The four elements are self-efficacy, including coping skills [13], motivation to be self-determined [14], to be able to feel a meaningfulness in activities, and the experience of impact on daily life [12]. Schulz and Nakamoto further tried to characterise the differences between the concept of health literacy and patient empowerment. Interestingly, from their perspective, patients can be empowered without being health literate, but according to their model this will result in inappropriate behaviour [12].

In 2015, Bravo et al. based on a mixed-methods study including a scoping review and qualitative interviews, proposed a concept map of empowerment in the context of people living with long term conditions. This map consists of five key components that they recommend to be addressed when developing empowering interventions. The first two components are related to the interaction between the patient and health professionals “ethos” and “moderators”. The three other components are related to the design of interventions i.e. intervention, indicator, and outcomes [9].

Recently, there have been several initiatives focused on how to create true person-centred health services, which calls for a new understanding of health-related empowerment in relation to people living with one or more chronic conditions. In 2016, WHO announced the Framework on Integrated People-Centred Health Services, which emphasises the need for “empowering and engaging people and communities” and “creating an enabling environment” [15]. In parallel, the European Commission has focused on promoting the research for solutions to better support chronic conditions and particularly multi-morbidity. This has mainly been done through their framework programmes but also through the establishment of initiatives like the European Innovation Partnership on Active and Healthy Ageing (EIP on AHA) and its six action groups (AGs) in 2012.

The EIP on AHA is a unique European forum with participation from both private and public partners including academia to join efforts in developing innovative solutions for the elderly. A significant aspect of their work is to identify how to engage and empower people [16].

In 2013, the EIP on AHA initiated a process to develop a model for identifying organisational readiness (the Maturity Model) for scaling up. This resulted in a 12 factor measure whose result can be expressed as a spider diagram which allows both initial assessment but also tracking progress in the implementation of integrated care in an organisation [17].

In the process of developing the Maturity Model, which later served as a template for the SCIROCCO project [18], it became obvious that a common understanding of empowerment amongst members of the EIP on AHA was needed, both within the AG B3 (focusing on integrated care) and across the five other AGs to be able to develop the items for the empowerment section 8 in the Maturity Model. In response to this need the study presented here was initiated as a “synergy project” by EIP on AHA to obtain a consensus-based understanding of patient empowerment.

**Fig. 1:**
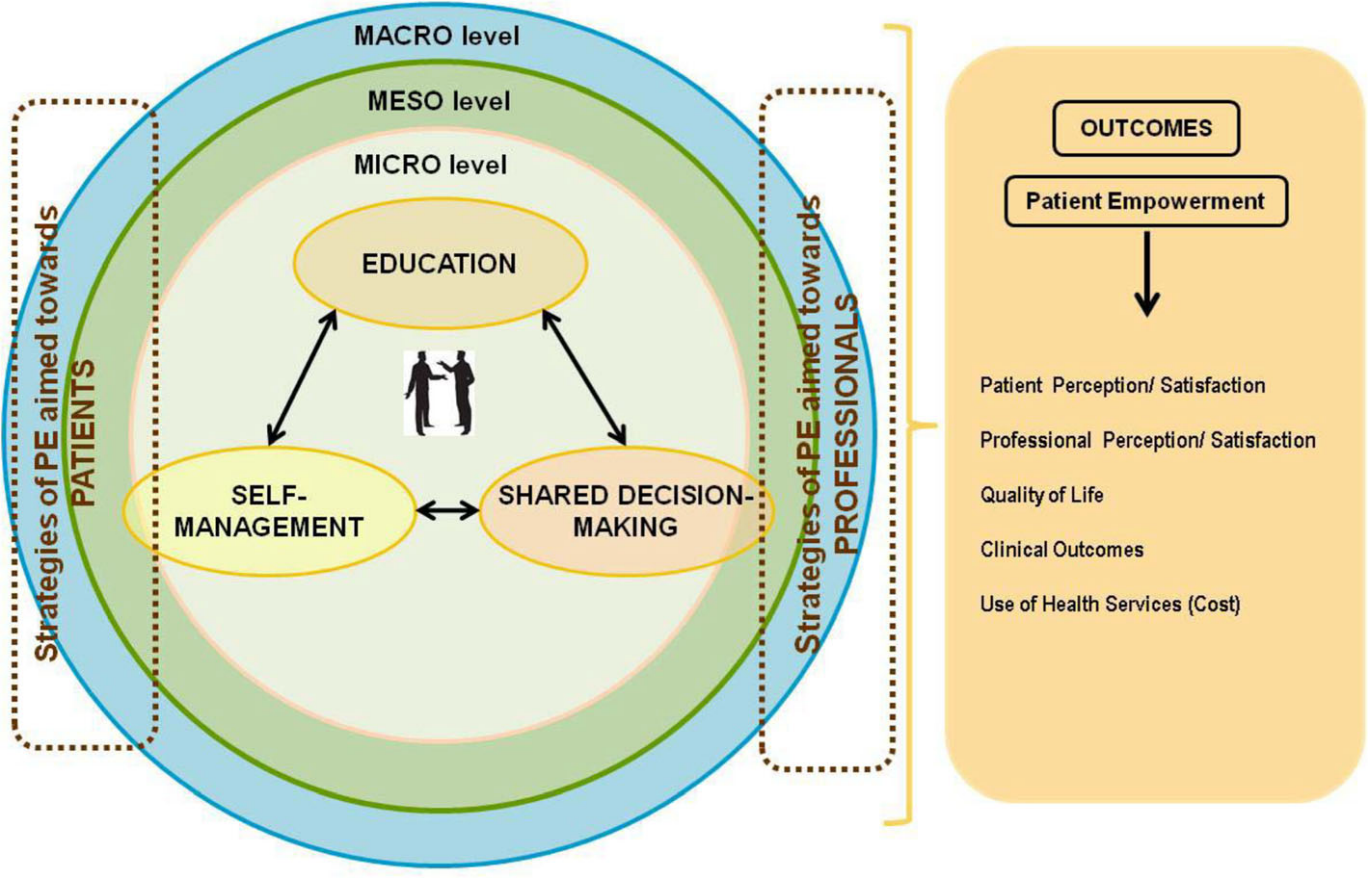
The EMPATHiE Framework. PE is an abbreviation for patient empowerment

It was decided to base the synergy project on the previous work of the EMPATHiE project funded by the European Commission [19]. The EMPATHiE project developed a conceptual framework for patient empowerment (Fig. 1) and based on this framework proposed a new definition of patient empowerment:

*“An empowered patient was defined as having control over the management of their condition in daily life, with the capacity to participate in decisions related to their condition to the extent that they wish to do so; to become “co-managers” of their condition in partnership with health professionals; and to develop the self-confidence, self-esteem and coping skills to manage the impacts of their illness on everyday living”* [19]*.*

The EMPATHiE framework clarifies the context in which their proposed empowerment definition stands. The three essential elements identified are education, joint decision-making, and self-management (Fig. 1).

The synergy project was initiated by sending out the EMPATHiE definition to a group of B3 AG members with particular interest in patient empowerment for commenting. Based on these inputs it was decided to change the patient-oriented definition to a more holistic understanding.

This aligns with WHO’s framework of Integrated People-Centred Health Services [15] as well as the “new wave of empowerment” which has influenced Europe with its focus on values, outcomes, professional leadership, and patient responsibility [9, 10].

As the next step of the synergy project the initial description of Health related empowerment was progressed through a Delphi inspired approach, where the proposal was sent out to all members of the AGs who had expressed an interest in the area of patient empowerment.

We here report on how we have developed and obtained consensus of a new understanding of health related empowerment amongst member of EIP on AHA action groups and discuss the possible implications of this new understanding in relation to how people can be empowered to manage their own health.

## Methods

The study was based on the Delphi process originating from the 1970’s [20]. This approach was chosen to obtain consensus as Delphi method is particularly suitable for areas of uncertainty and in need of structured communication [20]. According to the core principles of this method, a questionnaire was anonymously distributed to a group of experts in three sequential rounds. The questionnaire was distributed via email including a link to an online survey hosted by SurveyXact (SurveyXact, Rambøll Management Consulting). The questionnaire was sent to 122 members of the EIP on AHA network, who in relation to the renewal of their commitment in 2017 all signed up for participation in the patient empowerment synergy project.

**Fig. 2:**
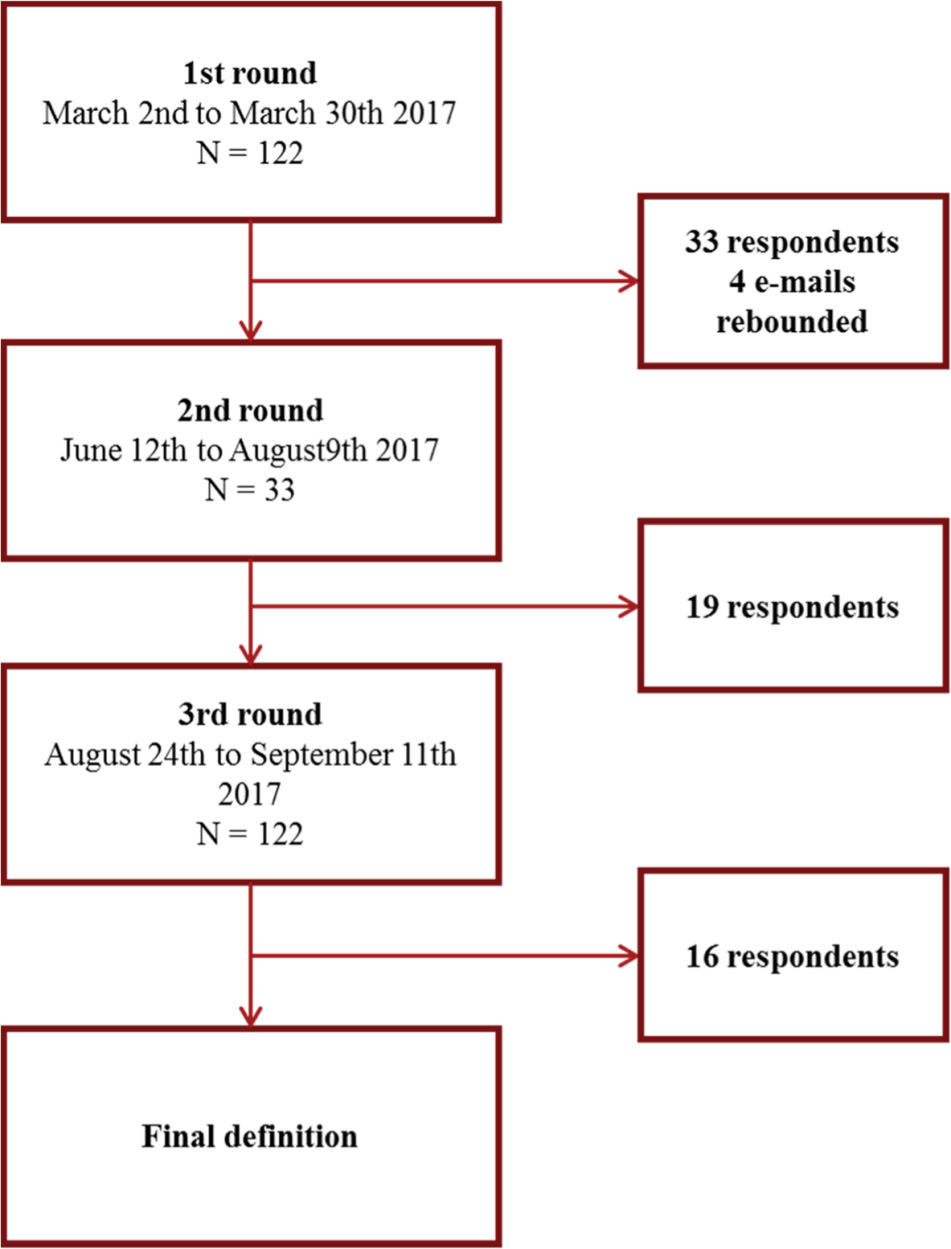
The Delphi process

The members are considered to represent expert organisations for two reasons; they belong to organisations with expertise in the field of active and healthy ageing and have through a reviewed commitment been admitted to the network. Secondly they have expressed interest for contributing to the field. The invited members represented a wide range of stakeholders including private and public care providers (28% of the total group of invited experts); national and regional administrations (12%); research and academia (33%); advocacy organisations for care professionals, older people, and patients (8%); large industries, insurance companies, and SMEs (15%); and clusters/organisations with specific interest in EIP on AHA (5%). An overview of the process is illustrated in Fig. 2.

The respondents were distributed as follows: 18% private and public care providers; 18% national and regional administrators; 33% research and academia; 21% advocacy organisations for care professionals, older people and, patients; 6% large industries, insurance companies, and SME’s; and 3% clusters/organisations with specific interests in EIP on AHA.

In the first round, we presented the suggested first version for a definition of “health-related empowerment” divided into nine statements (Table 1). The definition consists of two perspectives – an individual (statement 1–5) and an organisational approach (statement 6–9).s.

**Table 1:** Suggestion of an understanding of health-related empowerment based on an adaptation of the EMPATHiE Network patient empowerment definition

(1) Empowered individuals have control over the management of their condition in daily life. (2) They take action(s) to the extent that they wish to do so, to improve their health and the quality of their life. (3) A necessary prerequisite is that they are health literate, i.e. have the knowledge and skills, (4) and are conscious, self-aware and also determined to be involved (5) and if needed to adjust their health-related behavior. (6) Health-related empowerment interventions aim to equip individuals (and their caregivers whenever appropriate) with the capacity to collaborate in decisions related to the condition to the extent that they wish to do so; (7) to stimulate “co-management” of the condition; by creating a more equal power relation between the individuals and their formal and informal caregivers; (8) and to develop the individual’s self-confidence, self-esteem and coping skills, (9) enabling them to manage the physical, emotional and social impacts of their condition(s) in everyday life.	

The experts were then asked if they agreed or not with each individual statement, and, in the case they disagreed, to state the reasons for their disagreement. They were also allowed to comment even though they agreed in order to get a richer understanding of the various opinions.

In order to achieve a high number of answers, we sent an introduction with a link to the questionnaire to the members. Also, we sent reminders after 25 days of having sent the original emails. The participants did not receive any personal feedback, but all who completed the entire questionnaire in the first round (*n* = 33) were invited to participate in the second round.

The second round consisted of a revised version of the proposed definition for health-related empowerment, where some of the statements were modified based on the comments received in the first round. In particular, the original statements 1 and 2 were merged into a single statement and a new statement 9 was added. In the second round the previously described procedure was repeated.

The final definition obtained after the result of the second round was sent to all of the initial 122 experts. In the final and third round, the participants were asked whether they agreed to the proposed final definition in its full length and whether they had any comments in general and not by each statement.

Based on a discussion about the comments received in each round, the authors revised the statements. All the comments were considered in the analysis, even when the respondents agreed with the proposed statement as it was considered an opportunity to gain useful insights and information. There was not performed any text content analysis or more thorough coding of the responses. In the result section, we show selected quotes which emphasise important trends in the answers.

Before the investigation was initiated, the authors decided to consider an individual statement as valid when an agreement of 70% was obtained. This level was chosen based on the first authors experience with earlier unpublished Delphi studies and the expectation of receiving enough answers to calculate meaningful percentages of agreement. This choice was made, even though there is no consensus in the literature on the optimal way to decide the optimal level of agreement in a Delphi process [21]. After the first round it became obvious that all statements were agreed on between 70 and 94%. Even though agreement was reached in the first round, we decided to continue for three rounds [20]. We instead focused on how the comments during the rounds could help us obtain the highest degree of consensus to remove uncertainty and establish a common ground of understanding for the future work of EIP on AHA.

In the final round 16 out of the 122 invited responded to the questionnaire, and six commented on the overall proposed definition. No participants disagreed with the proposed final definition of health-related empowerment.

## Results

The responses to each statement are presented with statistics, comments and how the statement was modified. In the first round 33 of the 122 invited responded. In the second round only 19 of the 33 from the first round responded, whereof one participant only responded to first three statements.

### The individual perspective

*Statement 1: “Empowered individuals have control over the management of their condition in daily life”.*

Four respondents out of 33 (12%) disagreed. Six respondents contributed with suggestions. Three of the respondents suggested that the word “control” should either be replaced or the sentence modified with respect to individuals’ needs to be in control. One respondent wrote.

“It is difficult to accept that individuals even empowered, surely have control over all the different situations they encounter in their daily life”.

Another suggested that it is important to include the word motivation, as:

“Individuals’ empowerment towards their healthcare is also a function of their engagement (defined as their emotional adjustment to the disease condition and motivation to cope with it).”

Based on these comments, the word “control” was replaced with “able to and motivated” in the final definition.

*Statement 2: “They take action(s) to the extent that they wish to do so, to improve their health and the quality of their life”.*

Five respondents out of 33 (15%) disagreed. Six respondents commented on the statement. Two of the informants pointed to the fact that people are not always able to follow their wishes due to the social context or other external circumstances:

“The statement “to the extent that they wish to do so” implies they a degree of control over their actions; in some cases they may wish to do so but are unable to do so due to factors outside of their control e.g. financial difficulties, caring responsibilities, etc.”

“Not always; they need also a positive environment (services, resources, support) that facilitates these actions.”

Another comment was that the word “support” should be added and that they also should be capable to carry out what they wish to do.

Based on these inputs statement 1 and 2 were merged into a single statement and rewritten as follows for the second round:

*Statement 1 (former statement 1 and 2): “Empowered individuals are able to and motivated for taking action(s) in daily life to the extent that they wish to do so, to improve their health and well-being”.*

In the second round none of the 19 respondents disagreed or commented on this statement. It was then unchanged for the third and final round.

*Statement 3: “A necessary prerequisite is that they are health literate, i.e. have the knowledge and skills,”*

Ten out of 33 (30%) respondents disagreed with statement 3. Statement 3 was the statement which received most comments, with a total of 12 comments. Some of the respondents found that health literacy is not necessarily a prerequisite for empowerment. Others had difficulties with our attempt to embrace the full definition or understanding of health literacy with this very short statement. Thus, three different groups of opinions had to be included.

Those who disagreed:

“Not a necessary prerequisite but helpful. The common sense of people to be independent as long as possible and to live more years in good health feed the basic knowledge and skills for so. Thus, also the information and stimulus of a more informed and capacited society.”

“Health literacy requires empowerment but empowerment does not require health literacy.”

“Could it be that they do not have the knowledge - but the skill only? Do they have to have both?”

One informant who agreed to both statements 3 and 4 added this comment in relation to statement 4, which was taken into consideration when we discussed statement 3. Based on an input to statement 4;

“As stated above, I would stress the concept of individuals’ motivation. Individuals’ need to be motivated and need to have matured a psychological adjustment to their disease.”

The authors had originally found this coupling of empowerment with a need of knowledge and skills or even motivation or i.e. competence as included by the WHO definition in 1998 [22]. Based on the many inputs and the importance of this coupling, we consulted Ms. Kristine Sørensen from the former EU Health Literacy Consortium for advice. Ms. Sørensen was not a participant in EIP on AHA, but was invited to discuss this matter with the author group. Ms. Sørensen contributed to the rephrasing of the new statement two and suggested to add a new statement 9 at the end of the definition, which couples the organisation’s linkage to health professionals’ and individuals’ health literacy (see below). For the second round, we revised the statement as follows:

*Statement 2 (former statement 3):“A necessary prerequisite is that they are health literate, i.e. have the knowledge, motivation and competencies to manage their health and well-being.”*

In the second round, two out of 19 (10%) respondents disagreed with this revised statement. They were concerned that health literacy is not the only prerequisite and that it should be understood from a contextual perspective. After a thorough discussion, we decided to keep this text. Thus the new statement two was kept for the final round.

*Statement 4: “and are conscious, self-aware and also determined to be involved”.*

Two respondents out of 33 (6%) respondents disagreed with statement 4 in the first round. Four comments were made. One disagreeing respondent remarked:

“This seems to contradict the earlier sentence on ‘to the extent that...’ that may as well be deleted. I assume there are many people that are not conscious and self-aware due to their condition that we still would like to empower? Determination, to really want something, I do agree with”

And also a comment from one disagreeing respondent:

“May be cognitively impaired and so not completely self-aware but nevertheless empowered”

We here found it appropriate to remove the term conscious to partly address the cognitive impairment. During the discussion in the author group it was realised that the word “involved” should be replaced with ‘co-management’, to emphasise that it is not only about involvement but being an active partner in the management of their own condition. Thus the statement reads:

*Statement 3 (former statement 4):* “*They are self-aware and determined to be involved in the co-management of their health,”*

In the second round one out of 19 (5%) respondents disagreed with the new statement 3, and commented:

“I do not think health literacy has anything to do with being “determined to be involved”. Suggest deletion after self-aware. A person might be very health literate but also chose not to be involved!”

The new statement 3 was changed slightly in the final version. We substituted the word “determined” with “can choose” to underline the individual’s ability to choose if he/she wishes to be involved.

*Statement 5:“and if needed to adjust their health-related behaviour.”*

Three out of 33 (9%) respondents disagreed in the first round, and four respondents commented on statement 5. Based on suggestions from both some of the disagreeing statements:

“Adjust - is ‘change’ a better word?”

“Health-related behaviour - should other non-health related behaviour be included i.e. reduce social isolation by participating in relevant activities”

And one of the agreeing comments:

“if they decide, to adjust their health-related behaviour”.

Thus, we changed statement 5 into:

*Statement 4 (former statement 5): “and able to adjust their health-related behaviour if meaningful for them.”*

By adding the “able” and the clause “if meaningful” the individuals’ freedom to choose is underlined.

In the second round, only one out of 18 respondents (6%) disagreed and commented, which was a reference to a former input. Since this has already been considered, no further changes were made in the second round and thus statement 4 was not changed for the third round.

### The organisational perspective

The organisational perspective in the definition of health-related empowerment relates to the context in which the individuals are involved.

*Statement 6: “Health-related empowerment interventions aim to equip individuals (and their caregivers whenever appropriate) with the capacity to collaborate in decisions related to the condition to the extent that they wish to do so;”*

Only one out of 33 (3%) disagreed with statement 6, and two respondents commented. Based on the only disagreeing comment;

“... the capacity to be in control in decisions ...”.

We added “able to” as it is not only a wish but also the organisations should be aware of the individuals’ capacities. Thus the new statement 5 reads:

*Statement 5 (former statement 6): “Health-related empowerment interventions aim to equip individuals (and their caregivers whenever appropriate) with the capacity to collaborate in decisions related to the condition to the extent that they wish and are able to do so;”*

In the second round three out of 18 (17%) disagreed, and a total of four respondents commented on statement 5:

“The condition or their situation?”

“I partially disagree: To obtain (health and/or functional) benefit, they could need to adjust their health behaviour also ahead the extent they wish.”

“To the extent that they wish to do so. I am not entirely sure about the need of this addition that relates to the individual. The aim of the intervention is to equip the individual...no matter the wish of the individual”

“Interventions should aim to equip individuals (and their caregivers whenever appropriate) with the capacity to take decisions.”

These comments were thoroughly discussed but it was found that a revision was not necessary based on a wish to keep the focus on the individuals’ own ability to express their own wishes.

*Statement 7: “to stimulate “co-management” of the condition; by creating a more equal power relation between the individuals and their formal and informal caregivers;”*

Seven out of 33 (21%) respondents disagreed with statement 7. The statement received seven comments.

which all reflected views regarding the power relation:

“Suggest deletion of “power””

“To stimulate self-management...”

“Co-management or co-ownership?”

“...by creating an equal power relation…”

“By creating a more equal power relation between the individuals and their formal and informal caregivers’ should be rephrased to reflect more patient-centred than merely equal.”

In response to these comments, we removed the word “power” so the new statement read:

*Statement 6 (former statement 7): “to stimulate “co-management” of the condition; by creating an equal relation between the individuals and their formal and informal caregivers;”*

Four out of 18 (22%) disagreed on this second version. Most comments were about the word “more” and in both this and the first round stated that the term “formal caregivers” does not cover all health professionals. We did not change this widely used term, which together with informal caregivers provides a more equal impression of the formal caregivers around the individuals. As a second reflection in relation to the removal of the word power, we changed from the word “equal” to “mutual” to further reflect that it is not about a power structure but a collaborative effort. We also changed from the word “stimulate” to “enable” as this has a more open approach by enhancing the wish to create an enabling environment.

*Statement 6 (final):*

*“to enable co-management of the condition; through mutual agreement between the individuals and their formal and informal caregivers;”*

*Statement 8: “and to develop the individual’s self-confidence, self-esteem and coping skills,”*

Four out of 33 (12%) respondents disagreed, and four respondents left comments. In particular one respondents raised doubt about statement 8:

“I am unsure where self-confidence and self-esteem fits into the picture of one's health?”

Despite these comments it was decided to keep the original phrasing in the new statement seven for the second round:

*Statement 7 (former statement 8): “and to develop the individual’s self-confidence, self-esteem and coping skills,”*

In the second round only one respondent (6%) disagreed and commented:

“I do not think that self-confidence has anything to do with empowerment. They might be empowered in own health condition, but that does not have anything to do with their self-confidence or self-esteem. Only the coping skills - and perhaps Quality of Life (as studies indicate it does).”

Based on this argument being repeated from the first round and a reconsideration of the other initial inputs, the authors decided to change the word “self-confidence” into confidence and the word “self-esteem” was removed in the second round.

Thus, the final version of statement 7 reads:

*Statement 7 (final): “and to develop the individual’s confidence and coping skills,”*

*Statement 9: “enabling them to manage the physical, emotional and social impacts of their condition(s) in everyday life.”*

One out of 33 (3%) respondents disagreed with statement 9 in the first round, and six respondents commented. We decided to remove the “(s)” for the sake of simplicity. We also added “affects” to highlight that living with a condition is not a part time phenomenon but influences life 24/7.

Statement 9 was changed into:

*Statement 8 (former statement 9): enabling them to manage the physical, emotional and social impacts of their condition that affects their everyday life.*

In the second round only one respondent out of 18 (6%) disagreed to the new statement 8.

In the second round the new statement 9 was added as described above by input from Ms. Kristine Sørensen.

*Statement 9:* “*Empowering interventions foster the development of health literacy among staff and the people that they serve”.*

No one disagreed to this statement in the second round, and two comments were made. The statement was kept unchanged for the third and final round.

The revised version was sent to all of the initially invited stakeholders in the third and final round. This version got 16 responses with six comments where one marked “disagree” in order to call attention to his opinion

“‘Motivation’ is comprised in the first line of the definition. I would suggest to delete the second appearance (in the health-literate reference)”

Based on this comment, the final round led to only a minor adjustment by removing the word ‘motivation’ in statement 2 (former statement 3) to avoid a collision of the conceptual understanding of empowerment and that of health literacy. We also removed the parenthesis in statement 5 for the sake of simplicity.

The final definition of health related empowerment thus reads as presented in Table 2.

**Table 2:** Final understanding of health related empowerment

(1) Empowered individuals are able to and motivated for taking action(s) in daily life to the extent that they wish to do so, to improve their health and well-being. (2) A necessary prerequisite is that they are health literate, i.e. have the knowledge and competencies to manage their health and well-being, (3) they are self-aware and can choose to be involved in the co-management of their health, (4) and able to adjust their health-related behavior if meaningful for them. (5) Health-related empowerment interventions aim to equip individuals and their caregivers whenever appropriate with the capacity to collaborate in decisions related to the condition to the extent that they wish and are able to do so; (6) to enable co-management of the condition; through mutual agreement between the individuals and their formal and informal caregivers; (7) and to develop the individual’s confidence and coping skills, (8) enabling them to manage the physical, emotional and social impacts of their condition that affects their everyday life. (9) Empowering interventions foster the development of health literacy among staff and the people that they serve.	

## Discussion

This paper presents a new way to understand health-related empowerment, leaving behind the notion of the patient’s role and the previous understanding of control being the ultimate goal of empowerment [6]. This new understanding addresses the complexity of the health care system in which individuals have to navigate and make decisions in order to manage their own health. This new understanding of health-related empowerment also aligns with the WHO framework of Integrated People-Centred Health Services [15], by addressing the role of the formal and informal caregivers to create an enabling and empowering environment.

The strength of the proposed understanding of health-related empowerment is the dual perspective addressing both the importance of the interaction between the individual and the organisation and elaborating on this interaction, compared to the original definition of patient empowerment in the Ottawa Charter [6]. The new understanding also highlights the importance of health literacy in relation to empowerment as originally suggested in the EMPATHiE Framework (Fig. 1).

Our understanding is in contrast to the position of Schulz and Nakamoto, who considered empowerment and health literacy to be two distinct concepts. On the other hand, the conceptual understanding of empowerment suggested by Schulz and Nakamoto included self-efficacy, self-determination, meaningfulness and impact which all are implicitly embedded in the new understanding of health-related empowerment [12].

In this new definition of health-related empowerment, we no longer consider empowerment as being related to the individual’s role as a patient. This has been a necessary step to move from the traditional top-down, hierarchical, medical model and over to acknowledging the individuals’ knowledge and competence to be active co-managers if meaningful to them.

In order to facilitate a paradigm shift towards an empowered individual, and an enabling environment where health professionals actively support the individuals, it is essential to understand the existing power structures that are enforced in the traditional health services system. In the following, we will discuss some of the key aspect, which in our view need to be understood and addressed to effectively support such a paradigm shift.

One perspective of power is when you make someone act in a way that they would otherwise not have done [23]. In a medical setting, patients are often expected to obey the doctor, whose power is manifested through his expert role. This keeps the patient in a submissive role, being dependent on the health professional. To enable the individual to make decisions and not feel that these are imposed on them health professionals need to behave in a more co-creative way, avoiding behaviour that supports their position of power.

A second perspective of power that is important to understand is how power can be exercised not only through making someone act contrary to their wish, but also through non-decisions and by limiting the scope of decision-making [24]. This understanding of power points to a problem not yet solved but addressed in our definition. Namely, that the decision of people in a health context is not only influenced by the health professionals’ potential ability to make the patient act contrary to what they would have done otherwise, but also that health professionals can exert power over the patient by limiting the options available for decision making. Often when a new medical treatment or surgical procedure is proposed the professional is only able (or willing) to present some of the essential facts, which limits the patient’s ability to take a fully informed decision.

By making the health professionals more aware that the information and advice they offer may restrict the individual’s choices, and by addressing the individual’s health literacy, it is possible to create a more enabling environment, widening the individual’s space for decision-making. With increased health literacy the individuals may be able to complement the information provided by the health professionals and thereby achieving more power over their situation in a decision-making situation. But as our new definition emphasises, it is important to recognise the individual’s freedom to rely solely on the health professionals’ advice. By opening up the individual’s space for decision-making, power is transferred from the health professional to the individual, by allowing the individual greater choice over their situation, and also the opportunity not to take action themselves.

A third perspective that is necessary to understand is the structural and ideological power that is exercised through discourses i.e. language [25]. Power is not only exerted through actual actions but also through language. Examples of this implicit power are using the word “patient” and thereby inscribing the role of the submissive, vulnerable patient on the individual or the doctor’s use of diagnoses and medical terminology that enforces his expert role or indeed verbal or non-verbal cues that are overtly disempowering for the recipient.

The achieved definition is characterised by changing the focus from a patient, which ideally has control and is involved in the management of his or her condition to an individual, who is a co-manager and strives for well-being.

The core principles of health-related empowerment are the self-awareness, the confidence, the coping skills, and the ability to take action and manage your own condition in collaboration with your formal and informal caregivers, in a way where personal belief and integrity will be respected by all.

This may help us to better understand how we can facilitate the on-going transformation from the more radical version of empowerment towards the new wave with its focus on values, clinical outcomes and patient responsibility [9, 10]. This transformation, which has taken place for more than a decade in Europe, may also have contributed to how our members of the panel have responded and suggested the removal of the word “control” from the EMPATHiE based definition in its transition into the here proposed understanding of health related empowerment.

This new perspective does not necessarily imply that the individuals are without control of their health, but rather that they through active choices and responsible behaviour can manage their health. This may include, when they are in need of support, to distribute their responsibilities to their formal and informal caregivers.

Here, the notion of Bravo et al. [9] that it is important to take the influence of ethos and the role of moderators into consideration when planning for health services as insufficiently addressing these two areas may negatively affect how shared decisions are made in a clinical setting. Both the way power is exerted by health professionals, as discussed above, as well as the influence of caregivers’ own values and preferences may limit individuals’ ability to express their personal wishes [26–28]. The Guided Self-determination method may be of advantage here as it helps health professionals to understand the individual’s values and preferences through a reflective process which also develops the individuals’ life skills [29, 30].

A particular problem relates to those who are marginalised and disconnected from the traditional health services. These, often vulnerable individuals, need to be included before they can be empowered. Our new description of health related empowerment has, with its individual and organisational perspectives, helped us to recently discuss how to support these often vulnerable marginalised groups and how to include them by taking advantage of technology [31].

From an organisational perspective there are, according to Bravo et al. [9] two additional key components for fostering empowerment; “intervention” and “outcomes” ‘. In relation to these two areas, our new understanding of health related empowerment may help to inform interventions which are based on the WHO framework of Integrated People-Centred Health Services [15] or the Epital Care Model [32], both possessing values and ideas which are complementary to our new description.

Our new definition supports Zimmerman’s notions about personal control, a pro-active approach to life and a socio-demographic understanding of the environment and by including the intrapersonal and behavioural components. We did not directly address the interpersonal component, but it is in a way included in the requirement of health literacy. Interestingly enough some of the key elements from Zimmerman’s perspective (i.e. the elements of control or mastering, the self-confidence, and the need to often be conscious) disappeared from the definition as a result of the Delphi process and as consequence of the participants’ inputs.

This underpins the differences between being empowered in a psychological or social context such as in politics or community work in contrast to the context of being an individual living with burdens of disease treatment.

In a social or network structure, where dependencies due to expert status amongst some of the actors, may influence the individual’s wish to give control to others. This situation may also sometimes result in an empowered action with outcomes that may not be the ‘right’ ones from a medical expert or best evidence perspective. In this context “well-being” in its widest sense is an important outcome.

### Strengths and limitations

The selected Delphi process followed the classical model with three rounds, in which a large group of experts had the opportunity to comment anonymously. The latter may be of high importance, as the invited participants came from a broad range of institutions across Europe. With the anonymity, there is no hierarchical influence and all could raise their voice. Therefore, we think that the result of this process is more democratic than one which would have taken place through meetings within the network. Also, it gave us the opportunity to get the voices from a diverse range of stakeholders, including private and public organisations, industry, and organisations representing patients. These different perspectives may not be captured when trying to develop concepts based on literature or conversations with expert groups at meetings.

A limitation to the study is that even though respondents included members of advocacy organisations, the study does not directly include people with a lived experience of chronic conditions. Future studies of empowerment in a health context should involve people living with chronic conditions to include the voices of those we aim to empower.

A strength, at least in a European context, is the involvement in the creation of the new understanding by active participants in EIP on AHA. This has ensured an anonymous voice to those who are active members and have insight into the area from either a practice or policy perspective. The EIP on AHA members also contribute to the European strategy work such as the development of the Blueprint for digital transformation [33].

Finally, a limitation to this study is the relatively low number of participants. Although we designed the questionnaire to be responded to as briefly as possible, especially in the third round, questionnaires are often skipped in busy workdays. The study may have had increased validity if the result also was presented at workshops around Europe. The electronic method of distribution may have limited the accessibility for some stakeholders, but it was distributed via email with a web platform allowing answers via web browsers. This should not have been a barrier to experts in the field.

## Conclusion

We propose a new understanding of the concept patient empowerment, by focusing on the individual as a co-manager with freedom to choose and focus on their own well-being; an understanding which also requires a shift in behaviour amongst health service providers.

This new understanding of empowerment in a health-related context addresses the need to understand individuals with a health challenge across health services, organisations and disciplines. The definition is intended to be used in the EIP on AHA across all AGs as one that has been derived on a consensus basis, involving representatives from the AGs.

As the empowerment definition results from a commissioned synergy project, we would hope that the result would be adopted by the AGs and serve to provide a platform for future work aimed to increase empowerment, and life-skills, ultimately resulting in people who are able to understand, interact and maintain a sense of well-being. In particular, the section 8 of the B3 Maturity Model could be revised to mirror this new proposal across the EIP on AHA AGs.

The proposed understanding may also serve as an instrument to clarify the aims of the WHO Framework for Integrated People-Centred Health Services from 2016 where the primary intention is to unlock communities and individuals at all levels.

## Abbreviations

AG: Action Group
EIP on AHA: European Innovation Partnership on Active and Healthy Ageing
